# Business disruptions from social distancing

**DOI:** 10.1371/journal.pone.0239113

**Published:** 2020-09-18

**Authors:** Miklós Koren, Rita Pető

**Affiliations:** 1 Central European University, Budapest, Hungary; 2 Centre for Economic and Regional Studies, Budapest, Hungary; 3 CEPR, London, United Kingdom; University of York, UNITED KINGDOM

## Abstract

Social distancing interventions can be effective against epidemics but are potentially detrimental for the economy. Businesses that rely heavily on face-to-face communication or close physical proximity when producing a product or providing a service are particularly vulnerable. There is, however, no systematic evidence about the role of human interactions across different lines of business and about which will be the most limited by social distancing. Here we provide theory-based measures of the reliance of U.S. businesses on human interaction, detailed by industry and geographic location. We find that, before the pandemic hit, 43 million workers worked in occupations that rely heavily on face-to-face communication or require close physical proximity to other workers. Many of these workers lost their jobs since. Consistently with our model, employment losses have been largest in sectors that rely heavily on customer contact and where these contacts dropped the most: retail, hotels and restaurants, arts and entertainment and schools. Our results can help quantify the economic costs of social distancing.

## Introduction

Social distancing measures are effective non-pharmaceutical interventions against the rapid spread of epidemics [1–4]. Many countries have implemented measures such as school closures, prohibition of large gatherings and restrictions on non-essential stores and transportation to slow down the spread of the 2019–20 coronavirus pandemic [5–8]. What are the economic effects of such social distancing interventions? Which businesses are most affected by the restrictions?

Past research has analyzed the efficacy of social distancing interventions on reducing the spread of epidemics using the 1918 Spanish Flu in the U.S. [1–3] and seasonal viral infections in France [9]. Our knowledge of economic impacts, however, is limited [10]. For this question, past data may be less relevant, as the importance of face-to-face communication has increased steadily in the last 100 years through urbanization [11, 12] and specialization increased in business services as well [13, 14]. Even if advances in information and communication technology made it increasingly possible to communicate with co-workers and customers without the need for physical face-to-face interactions, personal contacts are still inevitable in some industries [15, 16].

The starting point of this paper is the observation that many sectors rely heavily on face-to-face communication in the production process [17, 18]. We build a model of communication to understand how limiting face-to-face interaction increases production costs. Without social distancing, workers specialize in a narrow range of tasks and interact with other workers completing other tasks. This division of labor reduces production costs but requires frequent contact between workers. In the model, the number of contacts per worker is the most frequent in businesses where the division of labor is important. When face-to-face interaction is limited, these are exactly the businesses that suffer the most.

To measure business disruptions from social distancing, we turn to recent data on the task descriptions of each occupation [19], the precise geographic location of non-farm businesses in the U.S. [20], and customer mobility patterns [21]. We construct three groups of occupations. First, some occupations require face-to-face communication several times a week with other workers. Examples of these *teamwork-intensive* occupations include maintenance, personal care related occupations and health care professionals. Other occupations require frequent face-to-face contact with customers. Retail salespersons, social workers and waiters and waitresses are examples of such *customer-facing* occupations. The third group of workers may need to be in physical proximity of one another even if they do not communicate, for example, to operate machinery or access key resources. Examples of such occupations requiring *physical presence* are drivers and machine operators, especially in mining and water transport, where crammed working environments are common. With our occupation level measures, we aim to capture the jobs that can be performed less efficiently from home. We validate our indexes by using the American Time Use Survey (ATUS) [22], which directly asks about the possibility of working from home.

To study how the patterns of interaction have changed in the U.S. during the Covid-19 pandemic, we use customer mobility data from SafeGraph [21]. This dataset measures the number of visits to a business in a given month, as captured from several cell phone apps and made available to researchers in an anonymized form. We study how the reduced number of customer visits is correlated with changes in sectoral employment.

## A model of communication

When workers communicate with others, they can divide labor more effectively. Production involves sequentially completing tasks indexed by *z* ∈ [0, 1]. A single worker can do a range of tasks, but there is a benefit to specialization and division of labor [23, 24]. The labor cost of a worker completing *Z* < 1 measure of tasks is *Z*^1+ *γ*^/*γ*, where *γ* > 0 captures the benefits to the division of labor. As we show below, the higher the *γ*, the more specialized each worker will be in a narrower set of tasks. Without loss of generality, we normalize the wage rate of workers to one so that all costs are expressed relative to worker wages.

Once the range of tasks *Z* is completed, the worker passes the unfinished product on to another worker. This has a cost of *τ*, which can capture the cost of communicating and interacting across workers. After all the tasks are completed, another step of communication with cost *τ* is needed to deliver the product to the customer. This cost leads to the Marshallian externality that firms want to be close to their customers and customers want to be close to their suppliers [25, 26].

The firm will optimally decide how to share tasks between workers. The key trade-off is economizing on the cost of communication while exploiting the division of labor [24]. Let *n* denote the number of workers involved in the production process. Because workers are symmetric, each works on *Z* = 1/*n* range of tasks before passing the work to the next worker. Production involves *n* − 1 “contacts” (instances of communication) and there is an additional contact with the customer.

**Fig 1:**
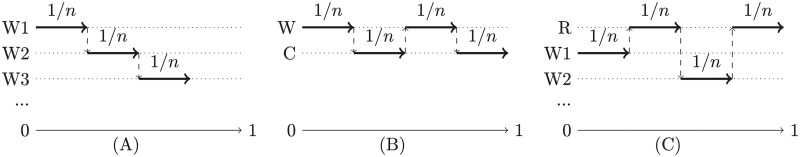
Patterns of interaction in the workplace. Horizontal movement represents production, vertical movement represents interaction. (A) Each worker W works on a range 1/*n* of tasks, passing work *n* − 1 times. (B) Worker W and customer C engage in frequent interactions. (C) Each worker W needs physical access to a key resource R.

Fig 1 illustrates the division of labor between workers. Horizontal movement represents production along a range of tasks (*Z* = 1/*n*), vertical movement represents interaction (*τ*). We note three potential interpretations of our model. First, when workers work in teams, they can efficiently divide labor among themselves (panel A). The benefit of a larger team is better specialization. Law firms, management teams, and IT service firms are prime examples of businesses where intensive communication leads to narrow specialization [27]. Second, communication may involve the customer (panel B). The benefit of more frequent interaction with the customer is a product or service that is better suited to their needs. Restaurants, beauty salons, personal and social services require such frequent interaction exactly because their service is so customized. Third, workers may need access to a key physical resource (panel C). In this case, even if they do not communicate, they may be subject to social distancing measures. For example, operators of machines, vehicle drivers or workers on an oil rig are all very much tied to a key resource to do their job. The key assumption behind all three interpretations is that frequent interaction increases productivity, whether happening between workers, between workers and customers, or between workers and machines.

The firm’s cost minimization problem can then be written as a function of the number of contacts alone,

$$\begin{array}{c} c\left(\tau \right)=\min_{n}n\tau +\frac{1}{\gamma }n^{-\gamma }, \end{array}$$ (1)

where total communication costs are *nτ* and production costs are *nZ*^1+ *γ*^/*γ* with *Z* = 1/*n*.

Given the strict convexity of this cost function, and ignoring integer problems, the first-order condition is necessary and sufficient for the optimum,

$$\begin{array}{c} n^{*}\left(\tau \right)=\tau^{-1/(1+\gamma )}. \end{array}$$ (2)

The number of worker contacts is decreasing in the cost of communication, expressed relative to worker wage. When the division of labor is important, *γ* is high, and the number of contacts does not depend very strongly on communication costs.

The total cost of producing one good can be calculated by substituting in (2) into (1),

$$\begin{array}{c} c\left(\tau \right)=\tau^{\chi }/\chi , \end{array}$$ (3)

where *χ* = *γ*/(1+ *γ*) ∈ (0, 1) measures the importance of division of labor. This unit cost function is the same as if workers and communication were substitutable in the production function in a Cobb-Douglas fashion. Indeed, *χ* captures the share of costs associated with communication and can be calibrated accordingly.

### Social distancing

We study the effect of social distancing, which reduces the number of face-to-face contacts to some exogenous value *N*. This may be mandated by government orders to close certain places of business or stay at home. But it can also be the result of voluntary social distancing in response to the risk of infection.

The optimal number of contacts without social distancing is given by Eq (2). Firms with n* > *N* are limited by social distancing. Their unit cost will increase to *c*′ = *Nτ*+ *N*^−*γ*^/*γ*, which is greater than the optimal cost,

$$\begin{array}{c} \frac{c^{\prime }}{c}=\chi \frac{N}{n^{*}}+\left(1-\chi \right){\left(\frac{N}{n^{*}}\right)}^{-\gamma }>1. \end{array}$$ (4)

The first term of the weighted average is less than one, representing a reduction in communication costs once the number of contacts declines. The second term is greater than one due to the fact that every worker has to complete a wider range of tasks than before, and they lose the benefit of specialization. Because n* is the cost-minimizing communication choice of the firm, the second term dominates, and production costs increase with social distancing.

## Data and methodology

To estimate the potential disruptions from social distancing, we need a measure of the importance of worker interaction (corresponding to *χ* in the model) and its change (captured by the ratio *N*/n*).

Let *ξ*_*o*_ denote an indicator equal to one if occupation *o* is interaction-intensive and zero otherwise. For industry *i*, *χ*_*i*_ = ∑_*o*_ *s*_*io*_ *ξ*_*o*_ measures the fraction of workers in affected occupations, with *s*_*io*_ denoting the employment share of occupation *o* in industry *i*.

We use the Occupational Information Network (O*NET) [19] to measure the characteristics of a given occupation, similarly to previous studies [15, 28–32]. The O*NET dataset contains detailed standardized descriptions on almost 1,000 occupations along eight dimensions. We focus on job characteristics that are related to recent social distancing measures, while prior work focused mainly on measuring offshorability of the given tasks [28, 29].

Social distancing interventions limit the interaction between people and regulate physical proximity between individuals. We thus focus on three related job characteristics based on work context and work activity described in O*NET. The first two indicators capture how communication-intensive the job is. Communication can be of two types: internal communication with co-workers (*teamwork*) or external communication directly with customers (*customer-facing*). The third indicator takes into consideration the possibility that workers may need to be in physical proximity of one another even if they do not communicate. We create an index that shows how important *physical presence* is to perform a given job. Table 1 details the specific O*NET indexes that contribute to each of our three measures. As social distancing measures only limit personal communication, for communication indexes, we require that the necessary face-to-face communication happens at least several times a week. Face-to-face meetings can often be substituted by more structured communication, for which working from home is not as disruptive. To allow for this possibility, we only classify occupations as teamwork-intensive or customer-facing where both emails and letters and memos are less frequent forms of communication than face-to-face meetings. This excludes most managers and certain business services. Similarly, for physical presence, we require at least a certain degree of proximity to other workers which corresponds to working in a shared office.

**Table 1:** Definition of social distancing indexes.

Index	Tasks	Context
Teamwork	Work With Work Group or Team	Face-to-face discussions several times a week & more often than emails, letters, memos
	Provide Consultation and Advice to Others	
	Coordinating the Work and Activities of Others	
	Guiding Directing and Motivating Subordinates	
	Developing and Building Teams	
Customer	Deal With External Customers	Face-to-face discussions several times a week & more often than emails, letters, memos
	Performing for or Working Directly with the Public	
	Assisting and Caring for Others	
	Provide Consultation and Advice to Others	
	Establishing and Maintaining Interpersonal Relationships	
Presence	Handling and Moving Objects	Density of co-workers like shared office or more
	Operating Vehicles, Mechanized Devices or Equipment	
	Repairing and Maintaining Electronic Equipment	
	Repairing and Maintaining Mechanical Equipment	
	Inspecting Equipment, Structures, or Material	

Each social distancing index (column 1) is created as an arithmetic average of the component indexes (column 2). To be classified an affected occupation, the average has to exceed 62.5 and the work context index has to exceed the threshold in column 3.

We aggregate the measures to 6-digit occupation codes (Standard Occupational Classification; 2010-SOC). We have information on the relevance of teamwork, customer contact and physical presence for 809 occupations in SOC 2010 codes.

**Fig 2:**
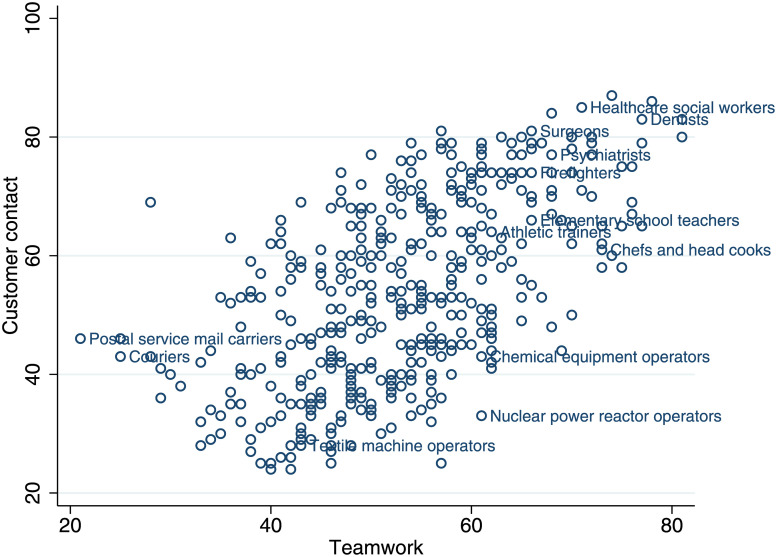
Teamwork and customer contact are highly correlated. Each circle represents an occupation. Teamwork and customer contact indexes are constructed as explained in main text.

Teamwork and customer contacts are highly correlated (Fig 2), but they are conceptually different. While all medical occupations require teamwork and customer contact, supervisors in general are working in teams but do not often communicate directly with customers. Machine operators and production workers in general are at the bottom of both of the distributions. As managers can substitute personal communication with emails, they are not considered in general as teamwork-intensive occupations according to our definition. Given the high correlation between the two types of communication, we often refer to *communication-intensive* occupations that are either teamwork-intensive or customer-facing.

**Fig 3:**
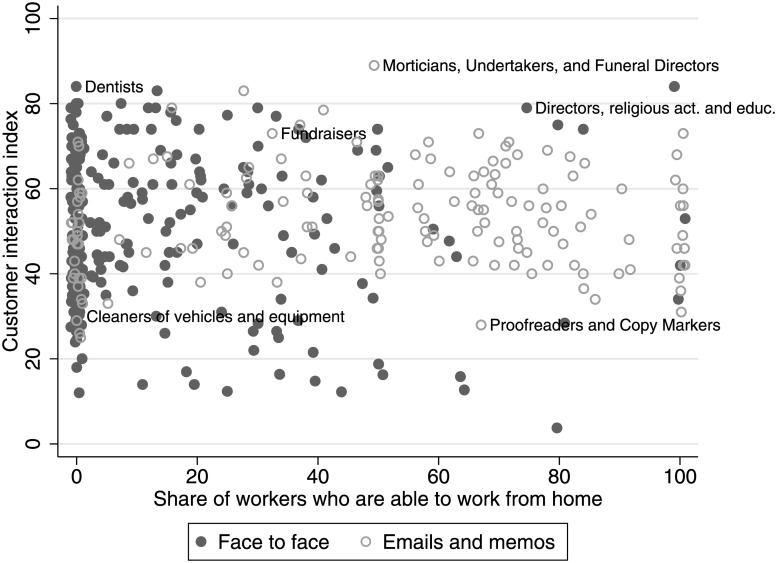
Workers in customer-facing occupations with face-to-face interaction can rarely work from home. Filled circles represent the occupations where face-to-face contacts are more important than emails and memos. Hollow circles represent the occupations where emails and memos are more important than face-to-face contacts. The indexes are constructed as explained in main text.

**Fig 4:**
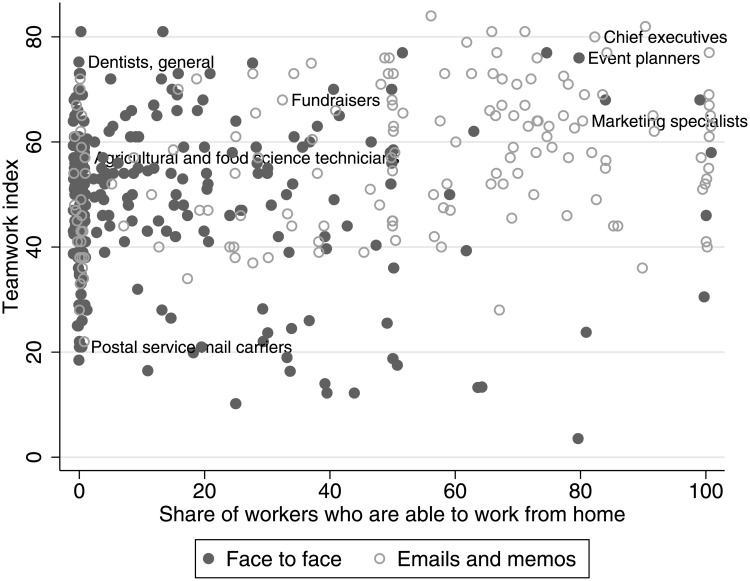
Workers in teamwork-intensive occupations with face-to-face interaction can rarely work from home. Filled circles represent the occupations where face-to-face contacts are more important than emails and memos. Hollow circles represent the occupations where emails and memos are more important than face-to-face contacts. The indexes are constructed as explained in main text.

With our occupation-level measures, we aim to capture the type of activities that require frequent face-to-face contact. Our assumption is that these activities cannot be effectively done from home. To validate this assumption, we use data from the American Time Use Survey (ATUS) [22], which directly asks workers whether they *can* work from home. Fig 3 plots our customer-intensity measure, for each occupation, against the share of workers who reported in ATUS that they can work from home (dark filled circles). Indeed, most customer-facing occupations have very few workers who can work from home. The pattern is different if we look at occupations that rely more on email, letters, and memos for customer communication (light hollow circles). The majority of these workers can work from home. Interestingly, for each degree of working from home (horizontal axis), there is sufficient variation in the importance of customer contact (vertical axis). The same patterns can be observed for teamwork-intensive occupations (Fig 4). This makes use conclude that dropping occupations primarily relying on email, letters and memos is sufficient to control for the potential to work from home.

With our validated occupation measures in hand, our next step is to calculate for each sector the share of workers whose job requires a high level of teamwork, customer contact, and physical presence. We use the same sectoral breakdown as the Current Employment Statistics (CES) [33]. As all the indexes are an absolute value running from 0 to 100, we use 62.5 as a cutoff to define a job to be teamwork-intensive, customers contact-intensive, or job that requires physical presence from the worker. The occupation structure of the industries are retrieved from the official industry-occupation matrix [34], we use the employment statistics by occupation-industry for February 2020.

Based on the share of relevant occupations in industry employment, the most teamwork-intensive sectors are, for example “Hospitals,” “Accommodation” and “Motion picture and sound recording industries.” In contrast, teamwork is not important in sectors like “Forestry and logging” and “Fishing, hunting and trapping.” Customer contact is relevant in sectors like “Hospitals” and “Retail”, while it is not relevant is sectors line “Truck transportation,” and “Forestry and logging.” Physical presence is relevant in sectors like “Truck transportation,” “Repair and maintenance,” mining in general, while it is not relevant in finance and information technology sectors.

“Hospitals” score high on all three measures because communication in health care teams and with patients is important, and doctors and nurses work in close physical proximity to others. We nonetheless remove this sector from the analysis because of its inevitable direct role in combating the epidemic which is not captured well in a simple model of communication.

To measure how the number of interactions has changed due to social distancing, we use data from SafeGraph [21], a data company that aggregates anonymized location data from numerous applications to provide insights about physical places. To enhance privacy, SafeGraph excludes census block group information if fewer than five devices visited an establishment in a month from a given census block group.

We use the Monthly Patterns file that captures the number of visits by mobile devices in the sample to more than 4 million points of interest (POIs) in each month. Each POI is assigned a specific address, including a ZIP code, and a 6-digit NAICS code. Because the pandemic hit different regions of the U.S. at different times, we use both the industry and the regional variation of customer mobility data. We aggregate monthly visits by 3-digit NAICS code and ZIP code. This enables us to measure by how much, for example, visits to clothing stores have declined in downtown Manhattan.

We measure the percentage change in the number of visits between February and May 2020. When the industry-ZIP cell received fewer than ten visits in either month or when visits data are missing in SafeGraph, we replace the change in visits with the average of the ZIP code.

To validate the customer mobility data, we check the location of sectors in the County Business Patterns (CBP) data for 2017 [20]. For a finer spatial resolution, we use the data tabulated by ZIP-Code Tabulation Areas. The CBP lists the number of establishments of a certain size for each ZIP-code and NAICS industry code. We estimate the employment of each industry in each ZIP code to be able to compute employment-weighted national averages of our statistics of interest.

Because establishment sizes are given in bins (e.g., 1–4 employees), we take the midpoint of each bin as our estimated employment (e.g., 2.5 employees). In small industries and ZIP codes, the Census omits some size categories to protect the confidentiality of businesses. We impute employment in these plants from the national size distribution of plants in the same NAICS industry. Our estimated industry-level employment is a very good approximation to official employment statistics [33]. The correlation between our estimates based on CBP and the employment reported in CES is 0.98.

### Counterfactual calculations

To gauge the magnitude of the effect of social distancing, we compute the effect of the decline in the number of customer-worker contacts. At the same time, we let the government introduce a proportional wage subsidy λ to help offset the costs from lower interaction. With this subsidy, the cost of labor will be (1 − λ).

We ask what level of λ would exactly compensate businesses for the communication disruption caused by social distancing. When interactions decrease, *N* < n*, production costs increase. We compute the subsidy λ, which, when given to every worker, would exactly offset the cost increase. This way the business would not have to fire any of its workers. The goal of this exercise is not to evaluate any particular employment support policy, but to get a sense of the magnitude of business disruptions.

Using the cost change in Eq 4, we can express the compensating labor subsidy of industry *i* in region *r* as

$$\begin{array}{c} \lambda_{ir}=1-\frac{1-\chi_{i}}{1-\chi_{i}N_{ir}/n_{ir}^{*}}{\left(\frac{N_{ir}}{n_{ir}^{*}}\right)}^{\gamma_{i}}>0. \end{array}$$ (5)

We calibrate $n_{ir}^{*}$ to match the number of customer visits to establishments of industry *i* in ZIP-code *r* in February 2020, assuming that these visits were optimal before the pandemic hit the U.S. The new number of visits, *N*_*it*_ will be calibrated to the number of customer visits in May 2020. The compensating wage subsidy increases in the importance of communication *χ*_*i*_ and decreases in the change in contacts $N_{ir}/n_{ir}^{*}$. The subscripts note that communication share is industry specific and the change in contacts is both industry and region specific.

To calibrate the importance of communication *χ*_*i*_, note that it is the cost share of communication, and can be correspondingly calibrated to the employment share of communication-intensive occupations in industry *i*. We then compute the compensating wage subsidy for each industry in each ZIP code using Eq 5. We report employment-weighted averages of this across sectors and across locations.

## Results

Table 2 displays the top five and the bottom five industries by 2-digit NAICS industries as sorted by the percentage of workers in communication-intensive occupations, excluding hospitals and clinics. Across industries, retail trade and accommodation and food services, arts, entertainment, and recreation have the highest share of communication-intensive workers, exceeding 35 percent. Information, transportation, production, professional, scientific, and technical Services and agricultural industries are less reliant on face-to-face communication. This heterogeneity across industries is important to understand the effect of social distancing measures.

**Table 2:** Retail, accommodation and restaurants are the most communication intensive.

Industry	Communication			
	Teamw.	Custom.	Overall	Presence
Retail trade	13	66	67	5
Accommodation & food services	8	50	51	1
Arts, Entertainment, and Recreation	12	38	40	2
Other Services (except Public Admin.)	12	30	33	12
Admin. & Support & Waste Manag.	17	24	27	7
…				
Wholesale Trade	8	12	15	12
Transportation and Warehousing	8	8	14	32
Prof., Scient., and Technical Serv.	5	10	12	1
Manufacturing	7	5	9	10
Agri., forestry, fishing & hunting	4	1	4	23

“Teamw.” and “Custom.” show the percentage of workers in teamwork-intensive and customer-facing occupations, respectively. “Overall” shows the percentage of workers in communication-intensive occupations that are either teamwork-intensive or customer-facing. It is less than the sum of the two indexes because some occupations rely on both types of communication. “Presence” shows the percentage of workers whose jobs require physical presence in close proximity to others.

Table 3 reports the results of regressing the log change in industry employment between February and May 2020 on our social distancing indexes. Each regression is estimated with unweighted ordinary least squares. Across the entire non-farm economy, employment has dropped by 13 percent (not seasonally adjusted) [33].

**Table 3:** Employment decline was sharpest in customer-facing industries.

	(1)	(2)	(3)
Customer-facing workers (share, [0, 1])	-0.418**	-0.463***	0.012
	(0.164)	(0.152)	(0.173)
Teamwork-intensive workers (share, [0, 1])	0.024	0.254	0.600
	(0.563)	(0.532)	(0.839)
Presence-intensive workers (share, [0, 1])	0.079	-0.051	-0.005
	(0.125)	(0.136)	(0.113)
Change in number of monthly visits (log)		0.185***	-0.119
		(0.063)	(0.131)
× customer-facing share ([0, 1])			1.021**
			(0.447)
× teamwork-intensive share ([0, 1])			0.332
			(1.500)
Observations	79	78	78
*R*^2^	0.187	0.302	0.435

Regression results of change in log industry employment between February and May 2020 estimated by ordinary least squares (unweighted). Explanatory variables in Column 1 are the shares of customer-facing, teamwork-intensive and presence-requiring workers. Column 2 controls for the change in log monthly visits to industry establishments. Column 3 interacts the change in visits with the share of face-to-face intensive workers in the two occupation groups. Robust standard errors are reported in parentheses. *p*-values are denoted by asterisk: * <.1 ** <.05 *** <.01. Sample excludes hospitals, clinics, and government establishments, as well as farming and fishing which are not present in CBP.

As Column 1 shows, the drop was larger in industries with a larger share of customer-facing workers. There is no significant correlation between the share of workers with teamwork-intensive jobs or the share of workers requiring physical presence to do their work and employment losses. In Column 2, we control for the change in log customer visits. Indeed, changes in customer visits are positively correlated with changes in employment (both dropping for most of our industries). In Column 3, we introduce interactions with the change in log customer visits (as a proxy for ln(*N*/n*)) and the share of communication workers (as a proxy for *χ*). As predicted by the model, the drop in customer visits has the largest effect on sectoral employment in sectors where the share of customer-facing workers is highest.

As we see from the regression results above, the largest decline in sectoral employment is in sectors where the share of customer-facing workers is highest. We hence use the share of customer-facing workers for the following analysis.

In the calibrated model, the social distancing that took place between February and May 2020 would be compensated by a 39.9 percent wage subsidy. The distribution of the compensating wage subsidy is, however, unequal across industries. Retail trade, where customer visits practically ground to a halt, would require a 234 percent wage subsidy. By contrast, the compensating wage subsidy in agriculture, transportation and manufacturing would be less than 2 percent (Table 4).

**Table 4:** The five most affected sectors require more than 14 percent wage subsidy.

Industry	Wage subsidy	Employment
Retail Trade	234	15,672
Arts, Entertainment, and Recreation	30.2	2,472
Accommodation and Food Services	26.1	14,394
Educational Services	22.2	3,828
Other Services (except Public Admin.)	14.5	5,941
…		
Wholesale Trade	1.8	5,934
Construction	1.1	7,639
Manufacturing	1.1	12,852
Management of Companies and Enterprises	1.1	2,447
Agriculture, Forestry, Fishing and Hunting	0.5	55
**Average**	**39.9**	**116,441**

“Wage subsidy” displays the percentage decrease in labor costs necessary to compensate businesses for the reduced number of customer-worker contacts. “Employment” is the February 2020 employment of the sector in thousands [33]. The last row shows the employment-weighted average wage subsidy. Table excludes hospitals, clinics, and government establishments which are not present in CBP.

## Discussion and conclusions

The main cost of social distancing in our model is insufficient division of labor. This mechanism is motivated by [23] and captures the same trade-off as [24]. Our contribution is specifying the cost function in such a way that can be easily mapped to the data.

More broadly, our argument is that frequent interaction increases productivity irrespective whether it is happening between workers, between workers and customers, or between workers and machines. In the main part of the empirical analysis, we focused only on the first two types of interactions, while we were silent about the third. But social distancing measures also affect sectors where workers need to be in physical proximity of one another even if they do not communicate, for example, to operate machinery or access key resources. This is relevant in sectors like “Mining, Quarrying, and Oil and Gas Extraction” and “Transportation” while it is not relevant in sectors like “Finance and Insurance” and “Professional, Scientific, and Technical Services.”

To a greater or lesser extent, all sectors will be affected by social distancing. Some sectors are hit by the intervention due to restricted face-to-face communication, others are hit due to restricting physical proximity of people. Some sectors are less affected across all dimensions. Examples include “Fishing, hunting and trapping,” “Printing and related support activities,” and manufacturing in general.

Our results are consistent with parallel research on the overall economic effects of the coronavirus pandemic using O*NET data. Recent research found that about 34 percent of U.S. jobs can be performed from home [15]. However, as our analysis points out, even among jobs that do not fall into this category, some are more at risk from social distancing than others. The share of workers working in close physical proximity to other people is similar to other recent estimates [31]. Workers in this group are found to be the most vulnerable across a wide range of socio-economic measures [30, 32]. We contribute to this work by (i) building a model to understand how social distancing measures affect the production, (ii) identifying three groups of occupations affected by social distancing and (iii) validating our model with customer visit and employment data.

We see three avenues for further research. The first concerns the interaction between sectors and regions. Whenever productivity in any business drops, this shock can propagate to its buyers and suppliers. The aggregate consequences of the epidemic will hence be modulated by input-output linkages between sectors, regions and countries [35–38].

The second and third directions concern the long-run response of businesses as they try to become more resilient to such shocks in the future. Whether the share of telecommunication remains large in the long run depends crucially on how easily it substitutes for face-to-face interaction. Previous work has found face-to-face communication to be more effective in high-intensity communication which is particularly helpful to overcome incentive problems in joint production [39, 40]. Data on internet flows suggests that telecommunication is not a good substitute for face-to-face meetings [41]. None of these papers discuss disruptions from social distancing measures.

Third, businesses may change their location in response to perceived threats and disruptions. Epidemics have a disproportionate effect on cities. So it is conceivable that in a post-pandemic spatial equilibrium (not modeled here, but see [18]), the agglomeration premium falls and firms find it less attractive to locate in cities. A poignant point of comparison is the increased threat of terrorism in major cities following devastating attacks on New York, Washington, London, Paris, Madrid, Moscow and Mumbai. The general conclusion about terror threat is that cities have remained resilient and a robust attractor of businesses [42, 43]. We speculate that epidemics and social distancing can be more detrimental to cities than terror threats, because they tear apart the very fabric of urban life. However, we have limited data to make further predictions.

## Supporting information

S1 Data Social distancing exposure by sector. The percentage share of workers in teamwork-intensive, customer-facing, and physical-proximity occupations within the industry. “communication_share” refers to the share of workers who are either teamwork-intensive or customer-facing. “affected_share” refers to the share of workers in any of the three occupation groups.
(CSV)
